# Calibrated Acoustic Leak Signatures in Pressurised Plastic Water Pipes: A Laboratory Analysis

**DOI:** 10.3390/s26144325

**Published:** 2026-07-08

**Authors:** Mohammad Reza Shekofteh, Kirill V. Horoshenkov, Edward John, Claire Gowdy, Andrew Blenkharn, Joby B. Boxall

**Affiliations:** 1 School of Mechanical, Aerospace and Civil Engineering, University of Sheffield, Sheffield S1 3JD, UK; k.horoshenkov@sheffield.ac.uk (K.V.H.); e.d.john@sheffield.ac.uk (E.J.); j.b.boxall@sheffield.ac.uk (J.B.B.); 2 Northumbrian Water Limited, Durham DH1 5FJ, UK; claire.gowdy@nwl.co.uk (C.G.); andrew.blenkharn@nwl.co.uk (A.B.)

**Keywords:** leak signal, acoustics, measurement, pressurised pipe, calibrated data

## Abstract

Despite decades of research, there is still a lack of calibrated data on acoustic leak signatures typical of common types of water supply pipes. This study addresses this gap by providing leak signatures recorded with calibrated, high-sensitivity accelerometers in a controlled laboratory environment. The study also investigates how different leak configurations at nominal static pressures of 2.8–4.2 bars influence the power spectrum of the pipe-wall acceleration. The results show a great variability, i.e., 5 orders of magnitude, in the power spectrum. The amplitude and shape of this spectrum depend on whether the leak is through a valve-controlled nozzle, hole directly drilled in the pipe wall, or a longitudinal or traverse slit. The coherence in the leak signals as a function of the distance between the accelerometers is determined and used to estimate the leak signal attenuation. Crucially, the results reveal that longitudinal slits, which represent the most common failure mode in plastic pipes, produce the weakest acoustic signals, making them difficult to detect and locate using standard acoustic equipment. It is expected that the calibrated data collected from this study will support high-fidelity computer simulations and development of better signal processing algorithms to predict and to detect hidden leaks in water distribution networks in the presence of background noise and high acoustic attenuation. The recorded data are made available to a wider community through a dedicated data depository.

## 1. Introduction

Public Drinking Water Distribution Systems (DWDSs) play a vital role in ensuring the safe and reliable delivery of drinking water. However, they are increasingly burdened by challenges such as ageing infrastructure, corrosion, pressure fluctuations, and contamination threats. One of the most pressing issues is leakage, which compromises operational performance and results in significant water loss and financial and energy/CO_2_ costs. Leaks can range from minor seeps to major bursts, with some being visible at the surface while others remain undetected, resulting in significant water loss over time [1].

Acoustic techniques are widely used for leak detection in pressurised water pipelines, since leaks generate structural vibrations that can be captured by accelerometers mounted on exterior access points such as service connections or hydrants [2]. The data recorded from such acoustic sensors can then be analysed to identify abnormal patterns indicative of leaks. Permanent acoustic monitoring DWDSs with accelerometers can be used to detect new leaks by analysis for change in the signal. It has been shown that this can even provide early detection of pipe cracks, as a crack’s formation creates extra vibration that raises and sustains the baseline noise level [3]. This sustained increase, isolated from daily fluctuations by analysing minimum noise levels in sliding windows, acts as an early warning before a crack becomes a burst. Temporary deployment of acoustic sensors is more common. Analysis of these signals for leak detection is generally hampered by a lack of knowledge of the characteristics of the sound source being sought, particularly for smaller leaks and multiple leaks, and is challenging in noisy urban environments. Hence, the most commonly used methods are time-delay estimation approaches for leak location such as cross-correlation between sensor pairs [2,4]. Despite the practical utility of these correlator-based methods, their localisation accuracy is limited, particularly in plastic pipes where high acoustic attenuation can lead to positioning discrepancies ranging from several metres to complete correlation failure. This can result in the excavation of ‘dry holes’ when the estimated location is incorrect [5]. These challenges motivate more in-depth laboratory studies to characterise the generation and attenuation of acoustic signals from different leak configurations to improve detection and localisation accuracy. This should be supported by carefully calibrated experimental data and physics-based acoustic models. Such combined evidence is essential for understanding how leak-induced vibrations are generated and propagate in realistic pipeline conditions.

### 1.1. Leak Source Characteristics

Acoustic methods rely on the vibrations or sound generated by water escaping through a leak in a pressurised pipe. These sounds and vibrations travel through the pipe materials and the water within it. The spectrum of leak noise measured with acoustic sensors, such as hydrophones and accelerometers, can vary depending on factors such as the water pressure within the pipe, the type of pipe, the surrounding soil, and the size and shape of the leak [6,7]. Although acoustic methods have proven effective for detecting and locating leaks in metallic water pipes, they are generally less effective in plastic pipes due to the higher attenuation of acoustic waves [8].

Surprisingly, there is a notable scarcity of calibrated data on the absolute spectra of acoustic leak signals, with many experimental studies reporting results in terms of normalised amplitudes, uncalibrated magnitudes, or relative scales. This scarcity is not a reflection of the importance of the topic, but rather a consequence of the technical challenges associated with implementing traceable sensor calibration and isolating background machinery noise within experimental facilities. Accurate, quantified measurements of leak signal spectra are essential for understanding attenuation effects and for progressing beyond leak detection towards leak characterisation, including the estimation of leak size, aperture geometry, and water loss rates, thereby providing information that may assist in planning repair strategies prior to excavation. While numerous studies have investigated acoustic leak detection, relatively few report calibrated measurements of sound pressure or pipe acceleration in physical units. For example, a rare experimental study [8] reported calibrated hydrophone data and mechanisms of leak noise generation in a 50 mm diameter water-filled plastic pipe with varying leak sizes (1–4 mm). Leak signals were recorded with hydrophones placed 50 cm from the leak. Results confirmed that turbulent jet flow is the main source of leak noise, with sound power measured 70–110 dB re. 1 mPa^2^/Hz between 70 Hz and 4 kHz. An empirical equation was provided to estimate the sound power level based on the pipe and leak hole diameters, leak exit velocity, and sound frequency. The sound power level scaled with the cube of hole diameter and increased with flow velocity, while pipe diameter also influenced the response.

Study [9] conducted experimental investigations into the feasibility of in-pipe acoustic leak detection in water pipelines using a tethered hydrophone (Bruel & Kjaer type 8103) positioned along the pipe centreline. In a laboratory recirculation loop pipe operating at line pressures of 1–3 bar, small leaks were simulated using a controllable valve (flow rates 0.082–0.266 L/s), and a large leak was generated using a fully open service connection (up to 10.74 L/s at 2 bar). Measurements taken approximately 0.5 m upstream and downstream of the leak revealed a distinct low-frequency broadband acoustic signature centred at 35 Hz for the small leaks and 45 Hz for the large leak. The leak signal was negligible upstream, strongest at the leak orifice, clearly detectable downstream for the small leaks, and noticeably attenuated downstream for the large leak due to outward radiation of acoustic energy. The authors showed that the acoustic amplitude from the leak increases strongly with hydraulic pressure (becoming reliably detectable above 1 bar) and highlighted that high-frequency components are heavily attenuated inside the pipe, leaving only low-frequency content useful for detection.

The study reported in [10] explored predicting leak flow rates in MDPE pipes using vibro-acoustic emissions recorded on a PCB 393B12 accelerometer and a Bruel & Kjaer type 8103 hydrophone, but the recorded spectra were presented in terms of an uncalibrated magnitude. Their experimental setup consisted of a 26 m looped pipe with an external diameter of 63 mm, incorporating a replaceable section to allow testing of various leak sizes. They found that leak flow rate and area had a strong effect on the recorded leak signal. The study in [11] investigated leak detection in buried plastic pipelines using KB12 (VD) piezoelectric accelerometer. While the authors presented calibrated time-series signal captures directly in physical units of acceleration (m/s^2^), their subsequent frequency-domain analysis relied on normalised spectral magnitudes. Consequently, this approach does not preserve the absolute physical units of acceleration or power spectral density, such as m/s^2^ or (m/s^2^)^2^/Hz, which limits direct quantitative comparisons of acoustic energy levels across different pipeline environments. Study [12] presented a benchmarking dataset generated from a laboratory-scale water distribution testbed featuring PVC pipes, comprising 280 measurements using both accelerometers and hydrophones. The study incorporated various leak types and background scenarios, such as fluctuating water demand and external traffic or machinery noise, to ensure realistic variability.

### 1.2. Propagation and Attenuation

Once the acoustic leak signature is generated at the source, its detectability is heavily governed by propagation and attenuation. Another typical issue is that the propagation and attenuation of leak-induced vibrations strongly depend on material properties of the pipe and its surroundings, influencing both signal amplitude and frequency distribution [8]. Using a pipe loop, a typical laboratory setup, e.g., ref. [10], complicates the propagation and attenuation of sound waves from the leak. The key factor influencing leak signal transmission is the pipe material and support as suggested in [5]. This study examined how pipe material affects leak noise transmission in a real water distribution system by creating artificial leaks (0.1, 1, and 5 L/s) in cast iron, asbestos cement, and polyethylene pipes. Leak noise was recorded using three accelerometers (10 V/g sensitivity): one near the leak, one upstream, and one downstream. The acceleration spectra were presented in the form of uncalibrated acceleration magnitude so that it is not possible to extrapolate from these data what was the actual amplitude of the acceleration recorded in these experiments. The results from the study showed that pipe material significantly impacts both the frequency spectrum and amplitude of leak signals. Cast iron and asbestos cement pipes allowed greater signal propagation and effective cross-correlation, while polyethylene exhibited high attenuation, reducing signal clarity and correlation accuracy.

Surrounding material and the pipe support method also significantly affect leak noise propagation in buried plastic pipes. Soil properties, particularly the shear modulus, have been shown to affect both the wave speed and attenuation of leak noise. The study in [13] developed an analytical model of the water–pipe–soil system, integrated with sensor dynamics to predict the frequency bandwidth of leak noise in buried plastic pipes. The study identified the surrounding soil as the dominant factor governing wave attenuation and propagation. It demonstrated that soft, sandy soils cause high attenuation which acts as a strong low-pass filter shifting the usable leak noise to lower frequencies and narrowing the bandwidth. In contrast, stiffer clay soils exhibit lower attenuation permitting a broader bandwidth that retains more high-frequency content. This study provides a practical method for estimating the critical leak noise bandwidth prior to field measurements and was validated against experimental data from test sites in the UK, Canada, and Brazil.

### 1.3. Geometric Effects and Modelling

Beyond material and environmental attenuation, the specific geometry of the leak aperture and its integration into numerical modelling frameworks play a critical role in advancing diagnostic methodologies. In recent years some studies, e.g., refs. [14,15,16,17], have focused on modelling the impact of different factors on leak noise signals generation and propagation, but all lack calibrated leak signature data. An analytical, numerical, and experimental study in [14] investigated how leak noise propagates in buried plastic water pipes. A 2D axisymmetric model was developed for the water, pipe, and surrounding soil, and experiments were conducted on two different soil types. The study found that pipe hoop stiffness and, critically, the soil’s shear modulus controls the wave speed. The system with stiffer soil also showed much lower attenuation, as only shear waves propagated into the soil, whereas in softer soil, both shear and dilatational waves removed energy from the pipe. Overall, the results demonstrate that soil stiffness strongly influences leak-noise propagation and therefore affects leak detectability in plastic water pipes. The study in [15] used the CFD analysis to assess the effect of leak geometry on internal pipe flow, comparing circular, square, and slot-shaped cracks of equal area. Their turbulence-based simulations showed that all geometries caused local pressure drops and velocity increases near the leak, but with geometry-dependent fluctuation patterns. Circular leaks produced symmetric single-peak profiles, square leaks resulted in broader regions with minor secondary oscillations, while slot-shaped leaks generated more complex and asymmetric signatures, highlighting the potential to infer leak geometry from flow behaviour. The study in [16] tried to simulate coupled fluid and acoustic behaviour for pipeline leak detection. The CFD module was used to model laminar water flow, showing that leaks cause a localised increase in velocity and a significant pressure drop at the leak location. Experimental measurements of acoustic noise from different leak sizes were then used to define a point source in the acoustic module. The simulations showed that sound pressure levels peak at the leak and decay rapidly along the pipe, with measurable attenuation over short distances, demonstrating that combined hydraulic and acoustic responses can be used to locate leaks and estimate their size. Study [17] investigated leakage-induced vibration signals using full-scale experiments on a DN200 ductile iron pipeline. The results showed that total vibration energy increases with internal pressure and leak size, while the characteristic frequencies are governed by leak geometry and remain pressure-independent. Higher pressures and smaller leaks shifted signal energy towards higher frequencies, supporting improved leak discrimination and cross-correlation-based localisation. While modern data-driven frameworks, such as those discussed in [18,19], are increasingly explored to classify pipeline anomalies using vibro-acoustic data and machine learning methods, the robust optimisation of these algorithms is inherently bounded by the availability of absolute baseline training data. Similarly, numerical predictive frameworks and finite element models—such as those implemented in COMSOL Multiphysics to simulate acoustic wave propagation and optimise sensor placement strategies, such as in [20]—rely entirely on accurately defined acoustic source strengths. Without establishing a baseline of fully calibrated, absolute physical quantities, numerical simulations risk under- or over-estimating real-world propagation limits.

This paper addresses the gap in calibrated leak acoustic data to support optimal placement of acoustic leak detectors. It presents controlled laboratory experiments with the primary aim of recording calibrated signals from a range of leak configurations under different hydraulic pressures and at multiple pipe locations. This information is essential for improving the understanding of the generation, propagation, and attenuation of leak-induced vibrations. Calibrated leak signal spectra are crucial for determining the maximum detectable range over which leak signals can propagate in plastic pipes and remain detectable by accelerometers of a given sensitivity. In addition, the comparison of leak generation methods (e.g., valve–nozzle configurations versus direct cuts) provides a practical insight into how laboratory setups may influence signal characteristics, highlighting that commonly used valve–nozzle arrangements can overestimate leak signal strength and may not fully represent real-world leak conditions.

This paper is structured as follows: Section 2 describes the laboratory experimental setup. Section 3 presents the signal processing of the leak noise data, including the calibrated acoustic measurements, coherence analysis of the recorded signals, and attenuation analysis describing the propagation of leak-induced vibrations at different locations along the pipe.

## 2. Materials and Methods

The aim of the experiments was to determine the absolute amplitude of the leak signal generated in the immediate vicinity of a leak and to estimate attenuation as a function of the distance along the pipe. To achieve this, the experiments were carried out to measure calibrated pipe-wall acceleration spectra near the leak and at a range of distances from the leak in a typical MDPE water pipe in the Contaminant Ingress into Distribution systems (CID) laboratory at the University of Sheffield [21,22].

The experimental setup employed a medium-density polyethylene (MDPE) water pipe, classified as PE80 SDR11, with a nominal outer diameter of 63 mm. PE80 is representative but is of a slightly lower grade than the most common plastic pipes installed in the UK, PE100. It is thus expected to have representative but extreme effects on the propagation of the acoustic signals. The pipe had a wall thickness of around 6.3 mm and a total length exceeding 60 metres arranged as a large oval loop with overall length and breadth of about 3 by 8 m. One section of the pipe was specifically designed for leak testing. This section was removable and replaceable to fit each leak aperture test scenario. To minimise background noise, such as from pumping, the facility was directly connected to large, elevated tank 5 stories above, which provided a maximum hydraulic pressure of approximately 4.2 bar. A pressure-reducing valve was fitted at the inlet to the system to control the pressure for each experiment, reducing it from this maximum. A system pump was used solely to fill the pipe loop from a water tank at the beginning of each test to ensure that there were no air pockets.

**Figure 1:**
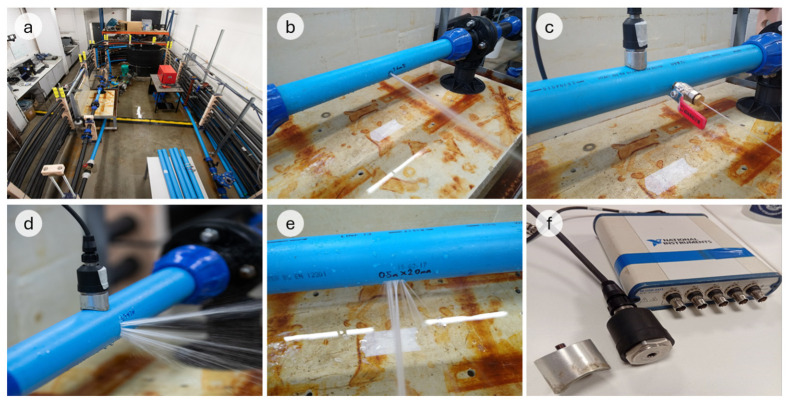
The pipe setup: (**a**) view of the pipe loop from above; (**b**) leak through a circular hole in the pipe wall; (**c**) leak through a valve and nozzle attached to the pipe; (**d**) transverse and (**e**) longitudinal leaks; (**f**) accelerometer with a curved-pipe saddle-mounting adaptor and a data acquisition device.

In order to generate a range of different acoustic signals, a range of leak sizes and shapes was introduced in the removable section. The selection of the leak aperture diameters and operating pressure levels was directly governed by the hydraulic capacity of the gravity-fed system. These included a ball valve fitted with interchangeable circular nozzles of varying diameters (1 mm, 2 mm, and 3 mm), as well as 1 mm and 3.6 mm circular holes directly drilled into the pipe wall. In addition, two slit-shaped leaks were created. Both were narrow rectangular openings measuring 0.5 mm × 20 mm, machined into the pipe wall. The first was a longitudinal slit aligned parallel to the pipe axis simulating a leak developing along the length of the pipe (a common failure mode in plastic pipes). The second was a transverse slit oriented perpendicular to the pipe axis (i.e., vertical in the laboratory setup) representing a leak across the pipe’s circumference (a common failure mode in cast iron pipes). While larger nozzle diameters (e.g., 4 mm and 5 mm) were initially considered, apertures exceeding 3 mm caused excessive localised pressure drops and hydraulic instability due to the finite discharge rate of the gravity supply. A minimum stable hydrostatic pressure of 2.8 bar could be consistently maintained across all tested configurations up to the 3 mm nozzle and slit apertures. Therefore, the test matrix was constrained to these boundaries to ensure rigorous, steady-state, and repeatable calibrated acoustic measurements. Figure 1 shows the experiment setup and the different leak shapes.

A graduated cylinder with an internal diameter of 14 cm (cross-sectional area of 0.0154 m^2^ and graduated with a 1 mm accuracy scale) was used to collect the leaking water to estimate the leakage flow rates. Table 1 shows the flow rates measured for different water pressures and leak apertures. The reported values represent the average flow rate obtained from at least two repeated measurements; in general, the repeated tests produced very similar results. The table also presents the estimated jet exit velocity, which was calculated by dividing the discharge flow rate by the aperture area. It varied within 30% between tests depending on the hydrostatic pressure in the pipe and the aperture dimensions. Direct openings in the pipe produced slightly lower flow rates for the same nominal size, suggesting flow resistance differences between valve and pipe-wall leaks.

High-sensitivity seismic accelerometers (Model 393B12, PCB Piezotronics, New York, NY, USA) shown in Figure 1 were used to measure the pipe-wall vibrations. This sensor choice aligns with the common practice among water utilities to use accelerometers rather than hydrophones as they can be easily attached to the pipe surface. The two used accelerometers in this paper were calibrated using a laboratory shaker and have sensitivities of 10,540 and 10,630 mV/g. Such high sensitivity and low noise make these sensors suitable for detecting vibration amplitudes associated with leak-induced acoustic signals. Data acquisition and instrument control were implemented in LabVIEW (National In-struments, Q3 2024), and the recorded data were analysed using MATLAB (R2023a).

**Figure 2:**
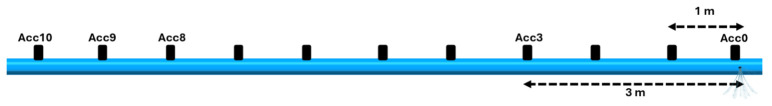
A schematic view of the pipe with the sensor locations.

For acoustic leak detection in plastic water pipelines the dominant signal energy and the usable coherence bands are consistently found at lower frequencies, typically well below 1000 Hz. As shown in the sensor’s official specification sheet, the device maintains the ±5% (0.4 dB) accuracy from 0.15 to 1000 Hz and a ±10% accuracy up to 2000 Hz (0.8 dB). It is a highly accurate sensor recording leak signals in which spectral power varies by a few orders of magnitude [23]. To ensure a rigid and secure coupling between the accelerometers and the curved exterior of the 63 mm MDPE pipe, custom-designed saddle-mounting adapters were used (see Figure 1f). In accordance with standard dynamic measurement practices to maintain high mounting stiffness, industrial petro-wax and a thin film of silicone coupling grease were applied at the contact interfaces. This standard approach ensures that the mounting assembly behaves as a rigid body extension of the pipe wall, preventing the introduction of localised compliance. Background noise in the pipe was measured under no-leak conditions, followed by recordings with a leak present in order to characterise the leak signals. Each test was conducted at least twice, with a duration of 30 s per recording and a sampling frequency of 8192 Hz. This time window and sampling rate allowed reliable capture of leak-related signals in the frequency range typical for leak noise. Two accelerometers in total were used to record the data. One was fixed as a reference, positioned at the leak location at the top of the pipe (12 o’clock position); see Figure 1c,d. The second sensor was moved along the pipe at discrete one-metre intervals, again placed at the top of the pipe. Figure 2 presents a schematic view of the pipe with the sensor locations arranged at 1 m intervals along the pipe. Throughout this paper, accelerometers are denoted as, Acc, followed by a number indicating their distance in metres from the leak (e.g., the accelerometer at the leak is Acc0, while Acc1 is located one metre downstream, and so forth).

To analyse the spectral characteristics of the signals, the time-domain accelerometer data (with unit of m/s^2^) were transformed into the frequency domain using the Welch periodogram method to estimate the power spectral density (PSD). The 30-s accelerometer signals were divided into 256-sample segments with 25% overlap. The frequency resolution was 32 Hz, meaning the first non-zero frequency bin occurred at ~32 Hz. A Hanning window was applied to each segment to reduce spectral leakage at the boundaries. Welch’s averaging procedure was then used to obtain stable PSD estimates for each accelerometer channel. This procedure was repeated for every test condition listed in Table 1, as well as for the background noise recorded in the absence of a leak.

## 3. Results

### 3.1. Leak Spectra

**Figure 3:**
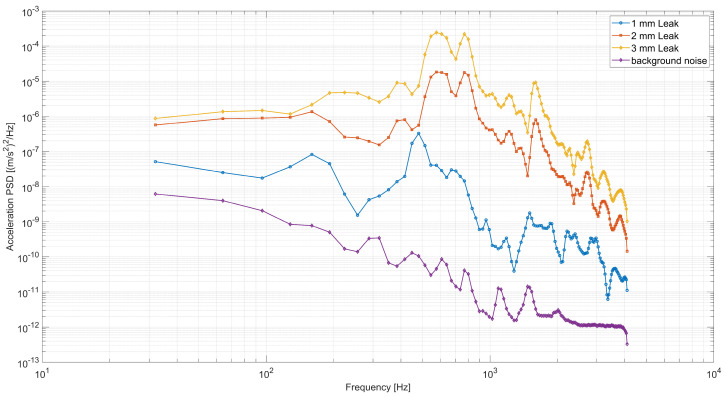
The effect of the leak hole diameter (with valve and nozzle) on the power spectral density for the pipe-wall acceleration measured at the leak location (Acc0) and hydrostatic pressure of 2.8 bar (Tests#2–4 in Table 1).

**Figure 4:**
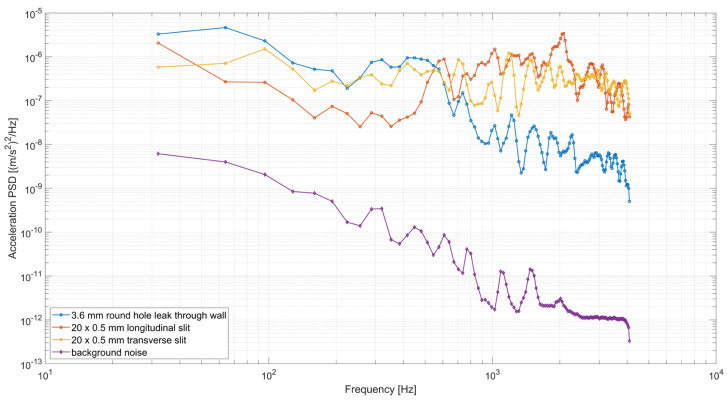
The effect of the leak aperture shape on the power spectral density for the pipe-wall acceleration measured at the leak location (Acc0) and hydrostatic pressure of 2.8 bar (Tests#6–8 in Table 1).

**Figure 5:**
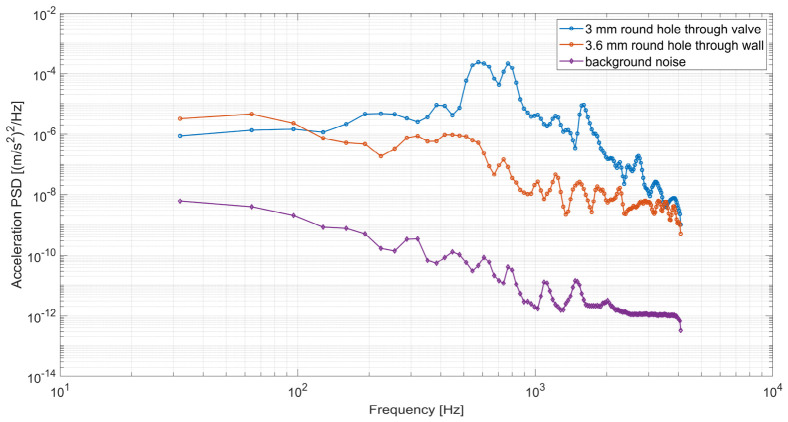
The effect of the pipe-wall acceleration recorded for the leak through the valve and nozzle and hole drilled directly in the pipe wall. The spectra are measured at the leak location (Acc0) for a hydrostatic pressure of 2.8 bar (Tests#4, 6 in Table 1).

**Figure 6:**
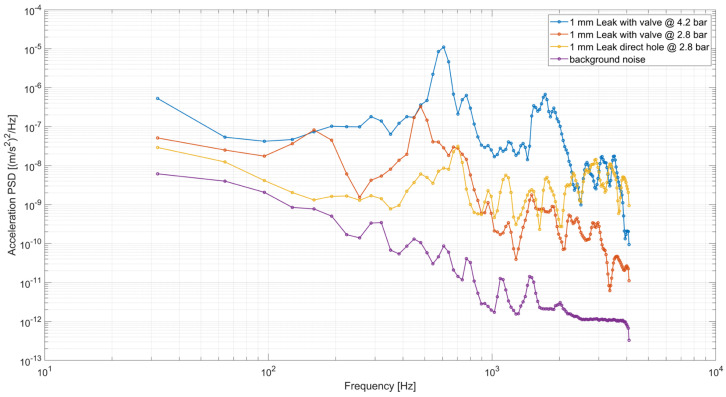
The effect of the hydrostatic pressure on the PSD generated by the 1 mm leak at the accelerometer position Acc0 (Tests#1, 2, and 5 in Table 1).

The results of the analysis detailed in the previous section are presented in Figure 3, Figure 4, Figure 5 and Figure 6 showing the calibrated power spectral density (PSD) of the acceleration in (m/s^2^)^2^/Hz. The actual accelerometer data are also available from the ORDA data depository [24]. Figure 3 demonstrates the effect of the leak hole diameter through a valve on the power spectral density for the acceleration measured at the leak location (Acc0 in Figure 2) and corresponding to 2.8 bar hydrostatic pressure (Tests#2–4 in Table 1). These results suggest that the leak through the 3 mm hole results in the highest amplitude of the pipe-wall acceleration with a clear peak between 500 and 900 Hz. According to Figure 3, the peak frequency for 2 mm is also between 500 and 900 Hz, while for the 1 mm leak, it is about 500 Hz. The frequency of this peak for the 3 mm hole roughly corresponds to that predicted through the Strouhal relation for the frequency of vortex shedding:

(1) $$f=S_{t}\frac{U}{D},$$

$S_{t}$ is the Strouhal number ($S_{t}\approx$ 0.18–0.26 for this flow regime and circular aperture shape), U is the flow velocity in m/s (jet velocity in Table 1), and D is the leak-hole diameter in m [25].

The broadband root mean square (RMS) acceleration, representing the total vibration energy over the full measured frequency range, was used to compare the severity of pipe-wall vibration induced by different leak geometries. The broadband RMS amplitude of the acceleration for the 3 mm hole was estimated at ${\bar{a}}_{s}\approx$ 233.7 mm/s^2^, approximately 2.5 orders of magnitude above the background noise (${\bar{a}}_{bn}\approx$ 0.7 mm/s^2^). Reducing the hole diameter from 3 mm to 2 mm (with valve and nozzle) reduced the RMS wall acceleration from 233.7 to 67.9 mm/s^2^, a factor of around 3.5 in amplitude, equivalent to about a 12-fold (slightly more than one order of magnitude) reduction in vibration power. However, the shape of the power spectrum remains similar for these two leak apertures. Further reducing the leak diameter from 2 mm to 1 mm (with valve and nozzle) decreased the RMS acceleration by a factor of about 10.8 (from 67.9 to 6.3 mm/s^2^), equivalent to about an order of magnitude in amplitude. In this case, the highest peak in the spectrum moves to around 500 Hz, which is not predicted with Equation (1) for that specific Strouhal number. Considering $S_{t}$ values around 0.1–0.2, the peak frequency for a 2 mm hole falls within 700–1100 Hz, as observed in Figure 3. The 1 mm case does not behave like a free jet; the flow is short and rapidly dissipated, producing much lower pipe-wall vibration compared with the 2 mm and 3 mm holes.

Figure 4 demonstrates the effect of the leak aperture shape on the power spectral density of the pipe-wall acceleration measured at the leak location for a 3.6 m direct hole against a slit-shaped leak with a similar leak orifice area (10.0 mm^2^ for slit vs. 10.18 mm^2^ for the 3.6 mm diameter hole). The PSD for the wall acceleration measured in the vicinity of the leak was relatively flat and does not show any pronounced peaks, in contrast to Figure 3 using valve and nozzle configurations. The broadband RMS amplitude of the acceleration produced by the leak through the 3.6 mm hole was approximately 26.7 mm/s^2^. The leaks through the longitudinal and transverse slits produced 48.5 and 37.3 mm/s^2^ broadband wall accelerations, respectively. The broadband wall acceleration produced by the leak through the longitudinal slit was approximately 30% higher than the transverse slit and about 80% higher than 3.6 mm direct hole despite the fact that the area of the apertures through which water was lost was nearly identical. However, in a broadly recognised leak signal frequency band (between 200 Hz and 800 Hz [9,13]), the 3.6 mm direct hole generated the 16.65 mm/s^2^ wall accelerations, which were higher than both slit-shaped leaks, with the transverse slit producing higher (15.44 vs. 11.8 mm/s^2^) acceleration amplitude than the longitudinal one. Hence, the common failure mode of longitudinal slits in plastic pipes produces the smallest signal, making them extremely challenging to detect and locate.

Figure 5 presents the difference between the acceleration power spectral density recorded from the leaks through the 3 mm hole in the valve and nozzle attached to the pipe and 3.6 mm hole drilled directly in the pipe wall. There was a very significant difference between the two spectra. Firstly, as mentioned earlier, there were no clear peaks in the pipe-wall acceleration spectra generated by the leak through the hole drilled directly in the pipe wall. Secondly, above 60 Hz the amplitude of the PSD reduces progressively with the increased frequency. Thirdly, in the case of the leak through the hole drilled in the pipe wall, the amplitude of the PSD was substantially lower than that recorded for the leak through the valve. The broadband acceleration produced by the two leaks was estimated at 26.7 and 233.7 mm/s^2^, respectively. In the frequency range between 200 and 800 Hz, this difference was close to 13-fold, 16.65 vs. 215.69 mm/s^2^. Figure 6 presents the PSD for a 1 mm leak produced by a hole drilled directly into the pipe wall and for a valve and nozzle, at 2.8 bar. While the difference is not as great as for the ~3 mm holes, considering frequencies below 1 kHz, the direct hole generated 2.5 times lower RMS acceleration than the valve–nozzle-controlled 1 mm orifice (2.46 mm/s^2^ compared to 6.23 mm/s^2^). These results show that leaks generated using a valve and nozzle (common in the literature) produce substantially higher pipe-wall vibration than leaks of comparable size formed directly through the pipe wall, indicating that valve- and nozzle-based leaks are not representative of real pipe failures from an acoustic perspective.

Figure 6 also presents the effect of the hydrostatic pressure on the PSD for the pipe-wall acceleration generated by the 1 mm leak through a valve and nozzle at the leak location. Increasing the pressure from 2.8 bar to 4.2 bar increased the broadband RMS acceleration by a factor of 5.5, from 6.3 mm/s^2^ to 34.9 mm/s^2^. This result is broadly consistent with previous studies, which indicate that higher pressures lead to increased vibration of leak signal [8,17].

**Figure 7:**
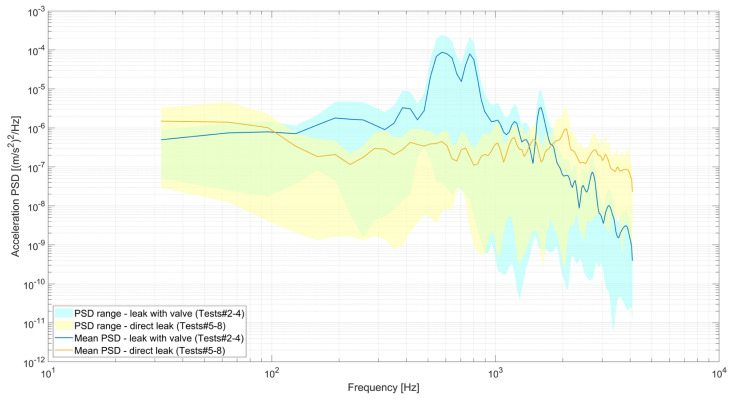
An illustration of the spread in the PSD related to the leak size and type. All the data shown are for the constant hydrostatic pressure of 2.8 bar (Tests#2–8 in Table 1) and correspond to the accelerometer position Acc0.

In summary, there is a very significant variation in the PSD for the pipe-wall acceleration generated by the leak types studied in this work. Figure 7 illustrates the range for the power spectral densities estimated for Tests#2–8, all conducted with a constant hydrostatic pressure of 2.8 bar, and at the leak location (Acc0). The blue zone in this figure is bounded by the minimum and maximum values of the estimated PSDs for the tests with valve- and nozzle-induced leaks (Tests#2–4), and the yellow shaded area is the range of the PSD for tests with direct leak in the pipe wall (Tests#5–8). The blue and yellow lines represent the mean acceleration PSDs for valve- and nozzle-controlled leaks and direct pipe-wall perforations, respectively. Leaks simulated through a valve generate significantly higher broadband pipe-wall vibration, with an average RMS acceleration of 140.6 mm/s^2^ compared to 33.5 mm/s^2^ for direct leaks, a factor of 4.2, and both shaded areas span an extremely wide amplitude range, covering 4 orders of magnitude. This is of a significant concern because it means that: (i) the detection range for these leaks can vary by several orders of magnitude; (ii) leak detection algorithms may fail to discriminate between the background noise and leak signals because these algorithms rely heavily on the knowledge of key features in the leak spectrum; (iii) there are likely to be problems to relate unambiguously the leak noise spectra with the size and shape of the leak. Also, there are significant differences between leaks simulated using a valve and nozzle (as in many previous studies) and those directly made in the pipe wall to represent a semi-realistic leak scenario. In essence, using a valve and nozzle to simulate leaks produces a very distinct peak in the spectrum, even when the flow rate is lower than that of a direct leak in the pipe wall.

### 3.2. Coherence Analysis

It is of interest to study the coherence between a pair of accelerometer readings to be able to understand the frequency range in which the acoustic attenuation can be estimated. Coherence provides a frequency-dependent measure of the linear correlation between two signals, and is defined as:

(2) $$C_{xy}\left(f\right)=\frac{{\left|P_{xy}(f)\right|}^{2}}{P_{xx}(f)P_{yy}(f)},$$

where $P_{xx}(f)$ and $P_{yy}(f)$ are the power spectral densities of the two signals, and $P_{xy}(f)$ is their cross-spectral power density. The coherence function ranges between 0 and 1, where values close to 1 indicate a strong linear relationship at a given frequency, and values close to 0 indicate weak or no linear dependence.

To evaluate the coherence between the accelerometer signals recorded at a range of positions along the pipe two representative datasets were selected: Test#4 (3 mm valve-controlled leak) and Test#6 (3.6 mm direct hole leak). Including both datasets allows for a comparative assessment of coherence behaviour under different leak conditions to ensure robust reference for reliable frequency-domain analysis. Coherence analysis was performed between the reference accelerometer located at the leak position (Acc0) and downstream accelerometers located at positions Acc1, Acc5 and Acc10 (see Figure 2). The coherence was computed using Welch’s method with a Hanning window of 2048 samples and 50% overlap, ensuring improved frequency resolution and statistical stability compared to the PSD estimation. The larger window length of 2048 samples was selected for coherence analysis to better resolve narrowband correlations between the accelerometer signals. The smaller window length of 256 samples was used for PSD visualisation to provide smoother spectral estimates. Rather than selecting a fixed frequency range a priori, the effective analysis band was determined directly from the data. A coherence threshold of 0.5 was applied consistently across all sensor pairs. From a practical perspective, maintaining a magnitude-squared coherence of $C_{xy}>0.5$ threshold ensures that the signal-to-noise ratio is above 0 dB, i.e., the signal power exceeds that of background noise. This defines the minimal acceptable threshold required for accurate leak localisation using standard industry equipment and algorithms. The analysis of the attenuation reported in the next section was carried out for the frequencies where coherence exceeded this threshold for all three sensor combinations. This intersection defines the frequency range where the signal-to-noise ratio is sufficient for us to assume that the two signals are for the leak.

**Figure 8:**
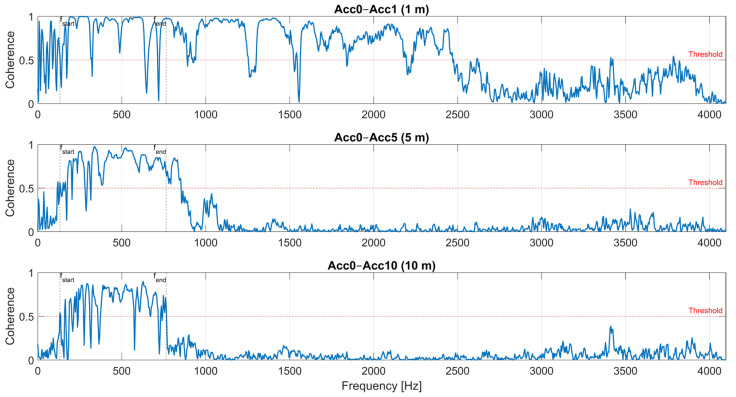
Coherence analysis for three pairs of sensors for Test#4 (3 mm leak with valve).

**Figure 9:**
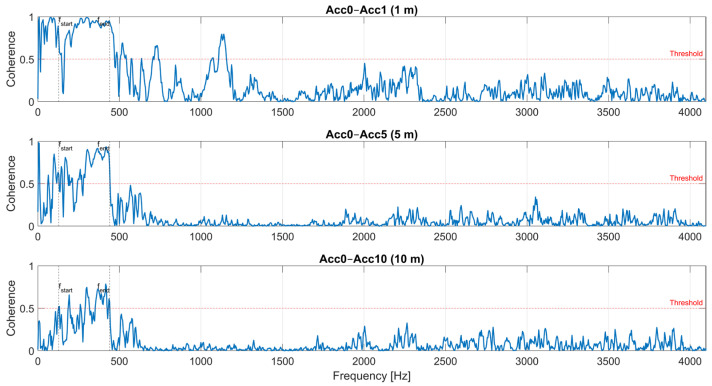
Coherence analysis for three pairs of sensors for Test#6 (3.6 mm direct hole).

Figure 8 and Figure 9 show examples of the coherence between pairs of sensors, with the red dashed line indicating the threshold ($C_{xy}=0.5$) as well as the start and end frequencies of the band for Test#4 and Test#6, respectively. For Test#4 (the case with the highest signal amplitude), a coherent propagation band of approximately 132–764 Hz was identified, corresponding to a bandwidth of 632 Hz. This band is interpreted as the dominant frequency range over which leak-induced vibrations propagate consistently through the pipe system, and it is subsequently used for attenuation and transfer function analysis. The observed fluctuations in coherence are most likely attributable to the wave interference in the experimental loop system caused by the propagation of the leak signal through the pipe supports, pipe bends, and fittings in the experimental setup (see Figure 1).

The coherence results for Test#6 indicate that the amplitude of the leak signal is quickly attenuated to become comparable with that of background noise. The frequency band in which the signals remain coherent is approximately 128–410 Hz (see Figure 9). At a distance of 10 m, the coherence between the signals at the Acc0 and Acc10 positions is highly oscillatory and mostly below the threshold, suggesting that the signal is no longer reliable for leak-detection purposes. This behaviour highlights the strong decay of leak-induced vibrations in the direct leak hole configuration (Test#6). In contrast, the valve-controlled leak produces higher signal energy resulting in more stable coherence over distance. These findings demonstrate that using a valve and nozzle to simulate leaks under laboratory conditions can lead to an overestimation of both the generated leak noise and its propagation range. For lower-energy leak scenarios, such as a direct hole, the signal degrades rapidly over a few metres, limiting the effectiveness of detection at larger distances.

### 3.3. Attenuation Analysis

**Figure 10:**
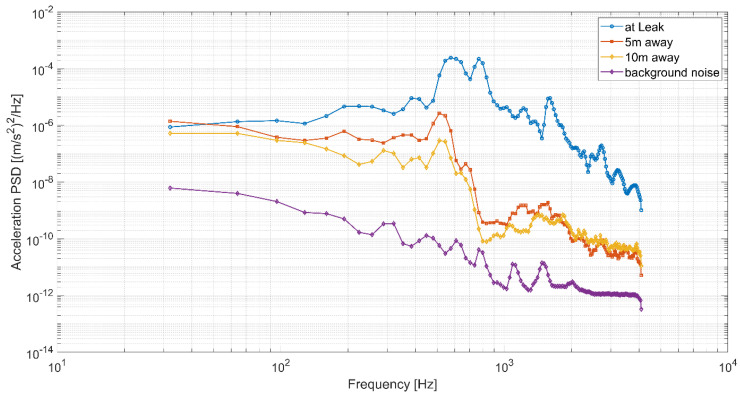
The effect of distance from the leak for the 3 m hole with valve and nozzle on the power spectral density for the measured pipe wall acceleration (Test#4).

Understanding acoustic attenuation is crucial because it affects how leak signals weaken over distance, impacting the detectability and localisation of leaks. Figure 10 illustrates how much sound power is lost with distance in Test#4 (3 mm leak). It shows three sets of acceleration spectra measured at the leak location, 5 m, and 10 m away from it. It also shows the background noise spectrum to provide an estimate for the signal-to-noise ratio. The results in Figure 10 show that leak-induced vibrations attenuate extremely rapidly, particularly above 550 Hz and within the first 5 m from the leak. In the frequency range determined in the coherence analysis (132–764 Hz), the RMS acceleration amplitude decreases by roughly a factor of 10, from 188.08 mm/s^2^ at leak location to 18.22 mm/s^2^ at a distance of 5 m. A further increase in the distance from 5 to 10 m away from the leak location reduces the amplitude by about another 2.6-fold. This supports previous findings that, although leak noise contains energy across a broad range of frequencies, the higher-frequency components attenuate very rapidly along the pipe, leaving the lower frequencies as the dominant contributors to the measured signal [13].

Theoretically, assuming the wave propagating in the pipe is a predominantly fluid-borne plane wave, the vibration energy decays approximately exponentially with distance as $E\left(\omega ,x\right)\sim e^{-2\gamma x}$, where x is the propagated distance along the pipe, ω is the angular frequency, and γ is the attenuation coefficient (Np/m). Since the PSD is proportional to the mean squared amplitude of the signal, it can be used as a statistically stable estimator of wave energy. Therefore, for two measurement locations at $d_{0}$ (reference) and d, the ratio of PSDs can be expressed as:

(3) $$\frac{P_{d_{0}}(\omega )}{P_{d}(\omega )}=e^{2\gamma (d-d_{0})},$$

$P_{d_{0}}(\omega )$ and $P_{d}(\omega )$ are the PSDs of the accelerometer signals at locations $d_{0}$ and d, respectively.

Taking the logarithm and expressing attenuation in decibels per metre gives:

(4) $$a(\mathrm{dB}/m)=\frac{10}{\left(d-d_{0}\right)}{log}_{10}\left(\frac{P_{d_{0}}(\omega )}{P_{d}(\omega )}\right),$$

Using the relationship between the exponential decay constant and decibel scale conversion, the attenuation coefficient in dB/m is related to γ as:

(5) a≈8.686γ,

In practice, however, the above formulation cannot be applied reliably over the full frequency spectrum due to the presence of noise, acoustic interference within the pipe loop, and loss of coherence at higher frequencies due to the high attenuation. Therefore, a direct broadband application of this formulation to the experimental attenuation data is not appropriate, as it includes frequency regions where the measured signals are either weakly coherent or dominated by background noise. To ensure physically meaningful attenuation estimates, the analysis was restricted to the coherence-derived propagation band of 132–764 Hz, corresponding to frequencies where the magnitude-squared coherence remained above the selected threshold ($C_{xy}>0.5$). The lower bound excludes low-frequency contributions associated with relatively high background noise and stronger acoustic interferences within the pipe loop, while the upper bound reflects the higher acoustic attenuation.

**Figure 11:**
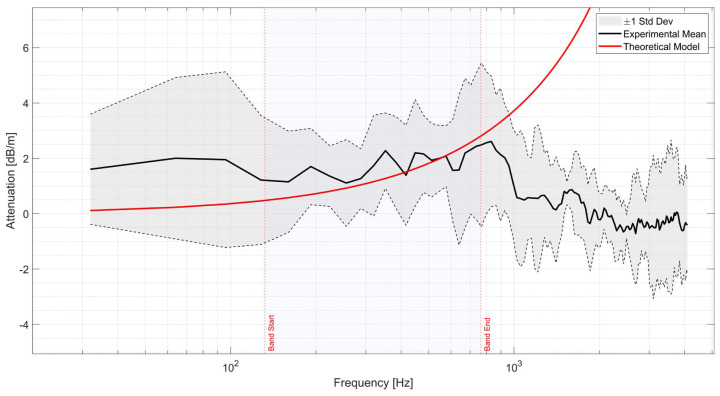
The range of acoustic wave attenuation estimated with Test#4 data for $d-d_{0}$ = 1, 2, …, 7 m, starting from Acc3 (3 m away from the leak).

Figure 11 shows the mean experimental attenuation based on the results from the experiments for Test#4 with accelerometer separations of $d-d_{0}$ = 1, 2, …, 7 m, starting 3 m away from the leak (Acc3 in Figure 2). Two key reasons to justify not using the closer sensor positions for the attenuation analysis are: (i) sensors positioned closer to the leak tend to record significantly stronger signals due to a limited dispersion and transformation of the acoustic leak signal in predominantly fluid-borne wave; (ii) the pipe between the leak location and 3 m away from it contains joints for the removable leak section that was designed to allow different leak scenarios and had T-junction installed to divert water in emergency situations. These structural elements introduce additional complexity to the acoustic propagation path affecting the leak signal measured at 1 m and 2 m more than at the farther sensor locations. Therefore, for all attenuation analyses data from 3 m away from the leak were used to provide a more consistent and representative spectral response while also helping to reduce complex wave interference effects. This multi-pair approach reduces dependence on any single sensor pair and provides a more representative estimate of the average attenuation behaviour along the pipe system.

Figure 11 also presents the theoretical attenuation predicted using Equation (5), where γ is calculated from the imaginary part of the complex wavenumber:

(6) $$\gamma =Im\left\{\omega /c\sqrt{1+\frac{2B_{f}/a_{p}}{Eh/a_{p}^{2}-\rho h\omega^{2}}}\right\}.$$

Here, c=1500 m/s is the speed of sound in water, $B_{f}$ = 2.2 GPa is the bulk modulus of water, $a_{p}=25.29$ mm is the inner radius of the pipe, E=1221+66i MPa is the complex Young’s modulus of the polyethylene pipe material assumed in the calculations [26], h = 6.31 mm is the pipe-wall thickness, and ρ=950 kg/m^3^ is the density of the polyethylene. It is important to note that this equation is very sensitive to the selection of the storage modulus, $E_{s}$, and the loss factor, η. Equation (6) is based on Equation (26) in Reference [27], assuming the surrounding medium has a sufficiently low density such as in the case when the pipe was surrounded by air in the reported experiments. The results presented in Figure 11 suggest that the theoretical value of the attenuation increases progressively with the frequency and significantly overpredicts the data from the pipe loop experiments.

In the selected frequency range the mean value of the measured attenuation is around 1.80 ± 1.72 dB/m. As shown in Figure 11, whilst the theoretical attenuation remains largely within the standard deviation of the experimental data, there is a clear discrepancy in the fundamental trends. The theoretical model predicts a monotonic increase in pure material attenuation with frequency. Conversely, the mean experimental attenuation plateaus and begins to decline above ~400 Hz. This decline indicates that the ratio of PSDs between the two sensors no longer represents pure material attenuation. Rather, it reflects an apparent attenuation that is heavily contaminated by constructive and destructive acoustic interference. This interference is an inevitable consequence of the 60 m pipe loop geometry, where acoustic reflections and standing waves are generated by downstream bends, coils, and the joints of the removable test section. Consequently, while the theoretical model characterises idealised propagation, the experimental data captures the complex, lab-specific apparent attenuation of a realistic closed-loop facility.

## 4. Discussion

The reported experiments investigated how leak size, shape, and pressure affect pipe vibrations, providing insights into leak-induced noise amplitude, propagation, attenuation, and the influence of flow rate. The data was recorded by high-sensitivity seismic accelerometers (Model 393B12, PCB Piezotronics). It was found that the acoustic signature of a leak commonly created in laboratories using a valve with a nozzle behaves very differently from that caused by a hole or crack in the wall of a pressurised plastic pipe. The difference in the power spectra generated by these leaks at the same static pressure can be as high as several orders of magnitude. For example, a 3.6 mm directly drilled leak hole can have a larger leak area and higher flow rate yet generate three orders of magnitude lower acoustic power than a smaller 3 mm valve-generated leak (Test#4 and Test#6 in Table 1 and Figure 5).

This behaviour is likely caused by increased turbulence and jet instabilities generated by the metal valve–nozzle geometry, which enhance fluid–structure coupling with the pipe wall and amplify the transmission of vibrational energy. Even the orientation of the slit (Figure 4) can have an order-of-magnitude effect on the leak spectrum. These findings suggest that laboratory leak simulators based on valves and nozzles may not accurately represent the acoustic characteristics of real pipe-wall leaks.

Analysis of attenuation in the frequency domain informed by coherence estimates, suggests consistency with the analytical model presented in [27] within the frequency range 132–764 Hz. However, outside this range, and particularly at higher frequencies, the experimental attenuation decreased, whereas the theoretical model predicted a monotonic increase. This discrepancy highlights that the ratio of PSDs between the two sensors no longer represents pure material attenuation. Instead, it reflects an apparent attenuation heavily contaminated by poor signal-to-noise ratios and constructive and destructive interference. The 60 m pipe loop inevitably introduces acoustic reflections, standing waves, and cross-talk from downstream bends, coils, and the joints of the removable test section. These facility-specific effects indicate limitations in the current experimental configuration for measuring absolute material damping. Further studies using fully straight pipe systems may help isolate pure propagation effects and provide improved datasets for model validation.

## 5. Conclusions

The main contribution of this work is the provision of calibrated acceleration spectra generated by leaks in a pressurised water supply pipe. These datasets have been made openly available [24] to support future numerical simulations of leak-noise generation and propagation in buried water pipes. Numerical models informed by calibrated acoustic source strengths can improve sensor placement strategies and help ensure that leak signals remain detectable above background noise.

The results demonstrate that leak geometry has a substantial influence on the generated acoustic signature. Leaks created using valve–nozzle arrangements can produce vibration levels several orders of magnitude greater than physically comparable holes or cracks in the pipe wall, highlighting the importance of using representative leak sources when developing and validating leak-detection models. In particular, the findings demonstrate that longitudinal slits, the most prevalent structural failure mode in plastic water pipes, generate the weakest acoustic signals among the tested geometries. This presents a major practical challenge for the water industry, highlighting that the most common leaks are inherently the most difficult to detect before they develop into catastrophic bursts.

The study also confirms the strong attenuation of leak-induced vibrations in plastic pipes, particularly for low-flow leaks, which may become indistinguishable from background noise over relatively short distances. The use of a pipe loop to estimate the attenuation is problematic because of reflections, standing waves, and acoustic interference from bends, coils, and the removable test section. While attenuation estimates showed agreement with analytical predictions over a limited frequency range, discrepancies at higher frequencies indicate that further investigation is required.

Future work should focus on more extensive laboratory and field measurements of realistic leak types under a wider range of operating conditions, together with experiments in simpler and more practical pipe configurations (straight pipes connected in a network). Such studies will provide more representative calibrated acoustic leak signatures and improve the accuracy of numerical models and data-driven leak-detection approaches.

## Figures and Tables

**Table 1:** Flow rates recorded for different hydraulic pressures and leak apertures.

Test Number	Leak Aperture Diameter	Pressure (bar)	Jet Exit Velocity (m/s)	Flow Rate (mL/s)
Test#1	1 mm with valve	4.2	19.6	15.4 ± 0.0
Test#2	1 mm with valve	2.8	18.3	14.4 ± 0.0
Test#3	2 mm with valve	2.8	15.3	48.2 ± 0.0
Test#4	3 mm with valve	2.8	16.9	119.3 ± 3.3
Test#5	1 mm direct hole	2.8	14.8	11.6 ± 0.4
Test#6	3.6 mm direct hole	2.8	15.1	153.9 ± 2.9
Test#7	Longitudinal slit	2.8	15.3	153.4 ± 2.2
Test#8	Transverse slit	2.8	17.4	174.0 ± 0.8

## Data Availability

The datasets generated during the current study are openly available in the University of Sheffield’s ORDA repository; see reference [24].

## References

[1] Leakage Routemap to Revolutionise the Reduction of Leaks. https://www.water.org.uk/news-views-publications/news/leakage-routemap-revolutionise-reduction-leaks.

[2] Farah E., Shahrour I. (2024). Water Leak Detection: A Comprehensive Review of Methods, Challenges, and Future Directions. Water.

[3] Zhang C., Lambert M.F., Stephens M.L., Gong J., Cazzolato B.S. (2020). Pipe crack early warning for burst prevention by permanent acoustic noise level monitoring in smart water networks. Urban Water J..

[4] Uchendu N., Muggleton J.M., White P.R. (2024). Wavelet-based and data-adaptive methods for time delay estimation in acoustic leak detection. Mech. Syst. Signal Process..

[5] Butterfield J.D., Collins R.P., Beck S.B.M. (2018). Influence of Pipe Material on the Transmission of Vibroacoustic Leak Signals in Real Complex Water Distribution Systems: Case Study. J. Pipeline Syst. Eng. Pract..

[6] Cody R. (2020). Acoustic Monitoring for Leaks in Water Distribution Networks. Ph.D. Dissertation.

[7] Martini A., Troncossi M., Rivola A. (2016). Leak Detection in Water-Filled Small-Diameter Polyethylene Pipes by Means of Acoustic Emission Measurements. Appl. Sci..

[8] Papastefanou A.S., Joseph P.F., Brennan M.J. (2012). Experimental Investigation into the Characteristics of In-Pipe Leak Noise in Plastic Water Filled Pipes. Acta Acust. United Acust..

[9] Khulief Y.A., Khalifa A., Ben Mansour R., Habib M.A. (2012). Acoustic Detection of Leaks in Water Pipelines Using Measurements inside Pipe. J. Pipeline Syst. Eng. Pr..

[10] Butterfield J.D., Meruane V., Collins R.P., Meyers G., Beck S.B. (2017). Prediction of leak flow rate in plastic water distribution pipes using vibro-acoustic measurements. Struct. Heal. Monit..

[11] Marmarokopos K., Doukakis D., Frantziskonis G., Avlonitis M. (2018). Leak Detection in Plastic Water Supply Pipes with a High Signal-to-Noise Ratio Accelerometer. Meas. Control.

[12] Aghashahi M., Sela L., Banks M.K. (2023). Benchmarking dataset for leak detection and localization in water distribution systems. Data Brief.

[13] Scussel O., Brennan M.J., de Almeida F.C.L., Iwanaga M.K., Muggleton J.M., Joseph P.F., Gao Y. (2023). Key Factors That Influence the Frequency Range of Measured Leak Noise in Buried Plastic Water Pipes: Theory and Experiment. Acoustics.

[14] Brennan M., Karimi M., Muggleton J., Almeida F., de Lima F.K., Ayala P., Obata D., Paschoalini A., Kessissoglou N. (2018). On the effects of soil properties on leak noise propagation in plastic water distribution pipes. J. Sound Vib..

[15] Ali S., Hawwa M.A., Baroudi U. (2022). Effect of Leak Geometry on Water Characteristics Inside Pipes. Sustainability.

[16] Chalgham W.R., Seibi A.C., Boukadi F. Simulation of Leak Noise Propagation and Detection Using COMSOL Multiphysics. Proceedings of the ASME 2016 International Mechanical Engineering Congress and Exposition.

[17] Yang X., Wang F., Fang H., Yu X., Li S. (2024). Numerical and experimental research on vibration signal characteristics of water pipeline leakage. Measurement.

[18] Sabbatini L., Esposito M., Belli A., Pierleoni P. Comparison of Signal Pre-processing and Machine Learning Modelling for Water-leak Detection Using Vibration and Pressure Data. Proceedings of the 2024 International Conference on Software, Telecommunications and Computer Networks (SoftCOM).

[19] Zhang C., Stephens M.L., Lambert M.F., Alexander B.J., Gong J. (2022). Acoustic Signal Classification by Support Vector Machine for Pipe Crack Early Warning in Smart Water Networks. J. Water Resour. Plan. Manag..

[20] Shekofteh M.R., Horoshenkov K.V., Gowdy C., Blenkharn A., Boxall J.B. Strategic Placement of Acoustic Sensors in Drinking Water Distribution Systems. Proceedings of the CCWI 2025—21st Computing & Control for the Water Industry Conference.

[21] Fox S., Shepherd W., Collins R., Boxall J. (2016). Experimental Quantification of Contaminant Ingress into a Buried Leaking Pipe during Transient Events. J. Hydraul. Eng..

[22] Collins R., Boxall J. (2013). Influence of Ground Conditions on Intrusion Flows through Apertures in Distribution Pipes. J. Hydraul. Eng..

[23] Model 393B12 Potted Accelerometer Enclosure Installation and Operating Manual. https://www.pcb.com/contentstore/docs/pcb_corporate/vibration/products/manuals/393b12.pdf.

[24] Acoustic Data and Code for the Leakage Experiments in the CID Lab, University of Sheffield.

[25] Testud P., Moussou P., Hirschberg A., Aurégan Y. (2007). Noise generated by cavitating single-hole and multi-hole orifices in a water pipe. J. Fluids Struct..

[26] NIST SRM 8456 Ultra-High Molecular Weight Polyethylene as a Reference Material for Dynamic Mechanical Analysis. https://www.tainstruments.com/pdf/literature/TA328.pdf.

[27] Muggleton J., Brennan M., Pinnington R. (2002). Wavenumber Prediction of Waves in Buried Pipes for Water Leak Detection. J. Sound Vib..

